# Platelet-primed interactions of coagulation and anticoagulation pathways in flow-dependent thrombus formation

**DOI:** 10.1038/s41598-020-68438-9

**Published:** 2020-07-17

**Authors:** Sanne L. N. Brouns, Johanna P. van Geffen, Elena Campello, Frauke Swieringa, Luca Spiezia, René van Oerle, Isabella Provenzale, Remco Verdoold, Richard W. Farndale, Kenneth J. Clemetson, Henri M. H. Spronk, Paola E. J. van der Meijden, Rachel Cavill, Marijke J. E. Kuijpers, Elisabetta Castoldi, Paolo Simioni, Johan W. M. Heemskerk

**Affiliations:** 1 Departments of Biochemistry and Internal Medicine, Cardiovascular Research Institute Maastricht (CARIM), Maastricht University Medical Centre+, P.O. Box 616, 6200 MD Maastricht, The Netherlands; 2 Department of Medicine, University of Padua Medical School, Padua, Italy; 3 Department of Protein Dynamics, Leibniz Institute for Analytical Sciences, ISAS, Dortmund, Germany; 4 Department of Biochemistry, University of Cambridge, Cambridge, UK; 5 Department of Haematology, Inselspital, University of Berne, Berne, Switzerland; 6 Department of Data Science and Knowledge Engineering, Maastricht University, Maastricht, The Netherlands

**Keywords:** Coagulation system, Platelets

## Abstract

In haemostasis and thrombosis, platelet, coagulation and anticoagulation pathways act together to produce fibrin-containing thrombi. We developed a microspot-based technique, in which we assessed platelet adhesion, platelet activation, thrombus structure and fibrin clot formation in real time using flowing whole blood. Microspots were made from distinct platelet-adhesive surfaces in the absence or presence of tissue factor, thrombomodulin or activated protein C. Kinetics of platelet activation, thrombus structure and fibrin formation were assessed by fluorescence microscopy. This work revealed: (*1*) a priming role of platelet adhesion in thrombus contraction and subsequent fibrin formation; (*2*) a surface-independent role of tissue factor, independent of the shear rate; (*3*) a mechanism of tissue factor-enhanced activation of the intrinsic coagulation pathway; (*4*) a local, suppressive role of the anticoagulant thrombomodulin/protein C pathway under flow. Multiparameter analysis using blood samples from patients with (anti)coagulation disorders indicated characteristic defects in thrombus formation, in cases of factor V, XI or XII deficiency; and in contrast, thrombogenic effects in patients with factor V-Leiden. Taken together, this integrative phenotyping approach of platelet–fibrin thrombus formation has revealed interaction mechanisms of platelet-primed key haemostatic pathways with alterations in patients with (anti)coagulation defects. It can help as an important functional add-on whole-blood phenotyping.

## Introduction

In the processes of haemostasis and thrombosis, platelet and coagulation pathways are tightly linked, with platelets both responding to thrombin and fibrin and, conversely, providing a phosphatidylserine-exposing surface on which high levels of thrombin and fibrin are formed^1–4^. Thrombotic and bleeding disorders are mostly related to a dysregulation of one of these pathways, for instance when linked to platelet or coagulation factor mutations, or to the (combined) use of antiplatelet or anticoagulant medication.

In the last decade, it has become clear that the accumulation of a platelet- and fibrin-containing plug or thrombus is instrumental to the onset of haemostatic as well as thrombotic events^5–7^. An important modifying factor in thrombus development is provided by the local blood flow^8,9^. This implies that, for adequate and comprehensive monitoring of the thrombotic process, measurements are to be performed under conditions of flow, preferentially recording both platelet and coagulation activation at the same time.

Recently, we have described a microspot-based whole blood flow test, allowing combined measurements of platelet activation processes at multiple different adhesive surfaces^10^. This work has revealed the roles of specific adhesive receptors in the thrombus-formation process in a shear rate-dependent way, i.e. glycoprotein (GP)Ib-V–IX (receptor for von Willebrand factor, VWF), GPVI and integrin α_2_β_1_ (receptors for collagens and collagen peptides), CLEC2 (receptor for podoplanin and rhodocytin), and integrin α_6_β_1_ (laminin receptor). Other flow measurements have demonstrated that the formation of a platelet thrombus is supported by the presence of tissue factor (TF)—acting via the extrinsic activation of factor (F)VII^11,12^. On the other hand, collagens and polyphosphates can trigger the intrinsic coagulation pathway through direct activation of FXII^13,14^. Phosphatidylserine exposure promotes the procoagulant role of platelets by markedly enhancing tenase activity (FX activation by FIXa and FVIIIa) and prothrombinase activity (prothrombin activation by FXa and FVa)^9,15^. Thrombin formation and clotting processes are restricted by natural anticoagulation pathways. The latter act by inhibiting activated coagulation factors (via antithrombin and tissue factor pathway inhibitor); and by inactivating FVa and FVIIIa through an (endothelial-derived) thrombomodulin and protein C pathway. Protein S is a cofactor for activated protein C (APC) in the inactivation of FVa and FVIIIa^16^. Currently, there is still a gap of knowledge of the relative contributions of the various coagulation and anticoagulation mechanisms in thrombus and fibrin formation under flow conditions.

In the present paper, we aimed to identify and characterize principal pathways that regulate the prothrombotic interactions between platelet and coagulation activation in flowing whole blood. For this purpose, we developed a microfluidics technique, allowing kinetic measurements of adhesion, activation and aggregation of platelets as well as fibrin formation during thrombus build-up. By triggering specific (anti)coagulation pathways, and by time-dependent measurements using multiple fluorescent probes, we generated time profiles of all these processes simultaneously. By analysing blood samples from patients with congenital defects in coagulation factors, we confirmed the pathophysiological relevance of alterations in thrombus formation parameters.

## Results

### Multiparameter assessment of platelet–fibrin thrombus formation on microspots in flowing whole blood

To simultaneously assess platelet activation and fibrin formation in microfluidic chambers, we extended an earlier microspot-based flow method, where only platelet responsiveness was studied^10^. For this purpose, we coated coverslips with microspots (*M*) consisting of platelet-adhesive proteins/peptides, as indicated in Table 1. The microspots were coated in pairs, where only the downstream spot contained TF (to locally trigger extrinsic coagulation); a procedure that prevents inter-spot cross-over effects^11^. Citrated blood was flowed through a microfluidic chamber over the microspots under conditions of two-step mixing with recalcification medium (Suppl. Fig. S1). Kinetic assessments were performed by capturing multicolour fluorescence and bright-field images per microspot every 2 min (t = 0–8 min). For each microspot, this resulted in 5 × 8 platelet, thrombus and fibrin parameters (Table 1). Time to first fibrin formation was recorded as an additional parameter (*P9*).

**Table 1:** Coding of microspots (*M*) and parameters (*P*) in whole blood thrombus formation, and curve characteristics (*C*) of thrombin generation.

*M*	1st Spot	Receptors	2nd Spot	Pathway
**Co-coating TF**				
*M1*	Control BSA	None	+ TF	FVIIa
*M2*	Rhodocytin + VWF	GPIb, CLEC2	+ TF	FVIIa
*M3*	Laminin + VWF	GPIb, α_6_β_1_	+ TF	FVIIa
*M4*	Collagen-III (VWF)^a^	GPIb, VI, α_2_β_1_	+ TF	FVIIa
*M5*	Collagen-I low (VWF)^a^	GPIb, VI, α_2_β_1_	+ TF	FVIIa
*M6*	Collagen-I high (VWF)^a^	GPIb, VI, α_2_β_1_	+ TF	FVIIa
*M7*	GFOGER-(GPO)n + VWF-BP	GPIb, VI, α_2_β_1_	+ TF	FVIIa
**Co-coating anticoagulant**				
*M8*	Collagen-I + TM	GPIb, VI, α_2_β_1_	− TM	Protein C
*M9*	Collagen-I + APC	GPIb, VI, α_2_β_1_	− APC	Protein C

*P*	Time (min)	Image type	Description	Unit
**Platelet parameters**				
*P1*	0–8	DiOC_6_	Platelet adhesion	%SAC
*P2*	0–8	AF568-annexin A5	Platelet PtdS exposure	%SAC
**Thrombus parameters**				
*P3*	0–8	Bright-field	Thrombus coverage	%SAC
*P4*	0–8	Bright-field	Thrombus morphology score	1–5
*P5*	0–8	Bright-field	Thrombus aggregation score	1–3
*P6*	0–8	Bright-field	Thrombus contraction score	1–3
**Fibrin parameters**				
*P7*	0–8	AF647-fibrin(ogen)	Fibrin deposition	%SAC
*P8*	0–8	Bright-field	Fibrin score	1–3
*P9*	0–8 (10)	AF647-fibrin	Shorter time to fibrin	11—*t* min

*C*	Curve characteristic	Unit		
*C1*	Shorter time to first thrombin	min		
*C2*	Endogenous thrombin potential (ETP)	nM min		
*C3*	Thrombin peak	nM		

1st Spots were located at upstream positions. Numbering of variables according to appearance in heatmaps.

^a^Binding VWF from plasma. *PtdS* phosphatidylserine.

### Accelerating role of tissue factor in kinetics of thrombus and fibrin formation

**Figure 1:**
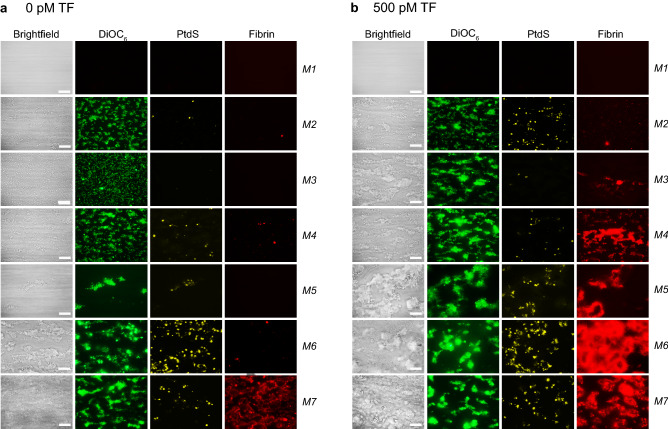
Simultaneous analysis of platelet deposition, thrombus phenotype and fibrin formation during whole blood thrombus formation under flow. Citrated whole blood from control subjects (*n* = 5–10) was supplemented with fluorescent labels to simultaneously detect platelet adhesion (DiOC_6_), procoagulant, phosphatidylserine (PtdS)-exposing platelets (AF568-annexin A5) and fibrin formation (AF647-fibrinogen). Blood samples were co-perfused with CaCl_2_/MgCl_2_ over indicated microspots, at a shear rate of 1,000 s^-1^. Microspot coding: *M1*, blocking buffer BSA; *M2,* rhodocytin + VWF; *M3,* laminin + VWF; *M4,* collagen-III; *M5*, collagen-I low; *M6,* collagen-I high; *M7,* GFOGER-GPO + VWF-BP; co-coating with 500 pM TF as indicated. (**A**) Representative bright-field and fluorescence images, taken from thrombi on microspots without TF (6 min). (**B**) Idem, from thrombi on microspots co-coated with 500 pM TF (6 min). Bars = 20 µm.

Based on the observation that co-coated TF enhances the coagulation process on collagen^11,17^, we determined how this altered the parameters of thrombus and fibrin formation for all microspots *M1–7*. In the absence of TF, platelet deposition and aggregate formation increased in the order of *M1* (BSA) < *M2* (rhodocytin + VWF), *M3* (laminin + VWF) < *M4* (collagen-III), *M5* (low-density collagen-I) < *M6* (high-density collagen-I) < *M7* (GFOGER-GPO + VWF-BP) (Fig. 1A). Accordingly, those microspots with high GPVI activation (*M6,7*) gave the largest platelet aggregates (*P1*, DiOC_6_) with highest phosphatidylserine exposure (*P2*, AF568-annexin A5) (Fig. 1A). In the absence of TF, only limited amounts of fibrin were formed, except for *M7*. However, with TF locally present, regardless of the type of microspot, the appearance of platelet aggregates preceded the formation of fibrin (Fig. 1B).

Complete data sets were then generated for microspots *M1–4,6* with different amounts of TF (co-coated 0–500 pM). Precise recording of times to first fibrin formation^18^ revealed a TF dose-dependent shortening of the coagulation times for *M2–4,6*, both at high (1,000 s^-1^) and low (150 s^-1^) wall shear rate (Suppl. Fig. S2).

**Figure 2:**
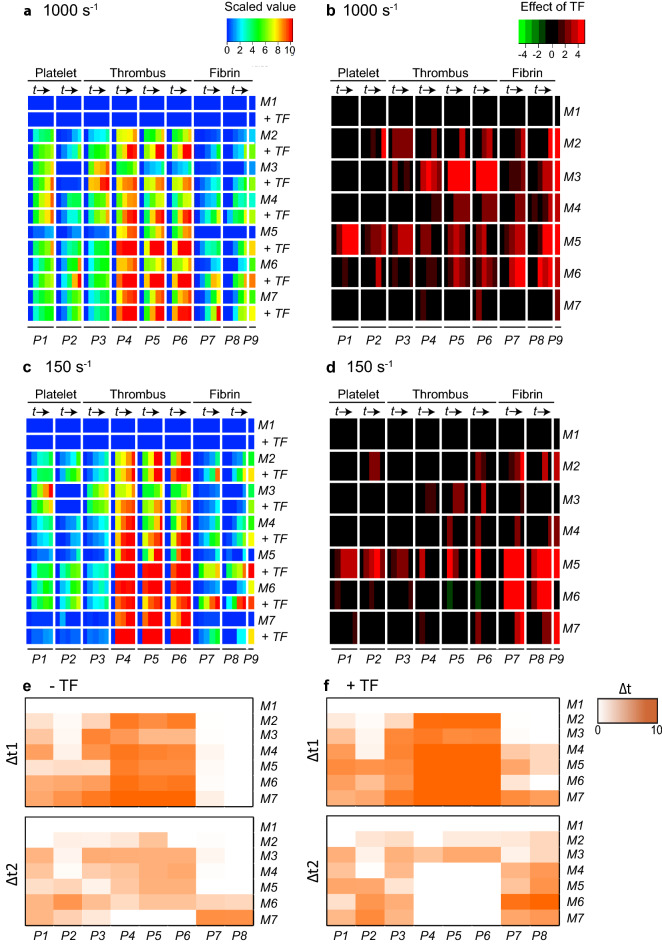
Surface-dependent enhancement by tissue factor of kinetics of platelet deposition, thrombus and fibrin formation. Whole blood with fluorescent labels was flowed over microspots *M1–7* with or without co-coated TF (500 pM), as for Fig. 2. Multicolour microscopic images were captured every 2 min (*t* = 0, 2, 4, 6, 8 min, →), and analysed for parameters *P1–9* (for coding, see Table 1). (**A**,**B**) Blood flow at high shear rate (1,000 s^-1^). (**C**,**D**) Blood flow at low shear rate (150 s^-1^). (**A**,**C**) Heatmaps of univariate scaled (0–10) values per parameter (*P1–9*) across surfaces with(out) co-coated TF (500 pM). (**B**,**D**) Subtraction heatmaps indicating relevant TF effects per parameter, filtered for changes outside range of mean ± SD. Means of flow runs, *n* = 5–10 subjects. Scaling was at 0–10 across all surfaces. (**E**,**F**) Differential increase in parameters *P1–9* (1,000 s^-1^) over intervals Δt_1_ = (0–4) min and Δt_2_ = (4–8) min per microspot without (**E**) or with (**F**) TF. Colour code indicates parameter increase (scaled 0–10 across surfaces, 8 min). Full data are given in Data File 1.

Evaluating the effects of high TF co-coating in more detail, we noticed a marked similarity between the shear rates of 1,000 s^-1^ or 150 s^-1^, which became apparent in scaled heatmaps (Fig. 2A,C) and in subtraction heatmaps (Fig. 2B,D). The CLEC2-dependent surface *M2* (rhodocytin) showed relatively high platelet parameters (*P1–2*) and thrombus parameters (*P3–5*) in the absence of TF, although TF still enhanced these parameters at high shear rate. For *M3* (laminin), TF was most effective at high shear rate, as demonstrated by the summed scaled effect of 84.03 versus 13.71 for high shear versus low shear, respectively (Suppl. Table S2). For *M4* (collagen-III), acting on platelets by weak GPVI activation, TF was only moderately active (small summed scaled effect of TF; Suppl. Table S2). On the other hand, for *M5* (low collagen-I, evoking moderate GPVI activation), TF enhanced all parameters including platelet deposition—likely as a limiting factor—, irrespective of the shear rate. The high-collagen surface *M6*, i.e. strongly GPVI-activating, caused maximal platelet adhesion and thrombus activation without TF present, while TF still enhanced the formation of fibrin. Microspot *M7* (GFOGER-GPO), again strongly GPVI-activating, behaved differently in that thrombus and fibrin formation were already maximal in the absence of TF (1,000 s^-1^). Yet, at low shear rate of 150 s^-1^, the presence of TF was required for appreciable fibrin formation on this surface. Of note, the addition of either a thrombin or FXa inhibitor completely abolished fibrin formation, regardless of the shear rate (data not shown)^19^. Taken together, these data pointed to a surface-dependent enhancing effect of TF on thrombus formation parameters, provided that coagulation activity and fibrin formation were not already maximal by prior platelet activation.

The capturing of time series of microscopic images allowed more precise analyses, in order to compare the kinetics of the various parameters. As a proxy approach to evaluate early and late events, we compared increases in parameters (scaled 0–10) during the first and second 4-min intervals of flow runs (Fig. 2E,F). This analysis showed that—across all surfaces and regardless of the presence or absence of TF—, platelet parameters (*P1–2*) mostly accumulated in the first interval but yet continued to increase, similarly to the growth of thrombus size (*P3*), while the other thrombus parameters (*P4–6*) had reached completion in the first interval. In contrast, again across all surfaces with(out) TF, parameters of fibrin formation (*P7–8*) were essentially restricted to the second time interval. This sequence of events is schematically modelled in Suppl. Fig. S3.

To assess the relative contributions of the two platelet parameters to the thrombus-forming process, we developed a mathematical regression model per microspot with(out) TF, which allowed us to determine which thrombus and fibrin parameters relied over time on the measured values of *P1–2* (Suppl. Fig. S3). Regardless of the type of surface, this analysis pointed to a consistently higher contribution of *P1* (65–85% of maximal prediction) than of *P2* (40–45% of maximal) for the thrombus parameters. The predictive power (7–27% of maximum) was lower for the fibrin parameters, due to the large time interval between platelet accumulation and (late) fibrin formation. On the other hand, the data did reveal a mechanism of thrombus formation primed by platelet adhesion and enhanced by thrombin formation, in which fibrin accumulates depending on TF and the platelet-activation strength of the surface.

To further substantiate this, we determined for 10 healthy blood donors how thrombus signatures correlated between the two high GPVI-activating microspots *M6* (collagen-I) and *M7* (GFOGER-GPO). Correlation analysis of all scaled values over time for the non-TF microspots gave highest positive correlations (*r* > 0.5, *p* < 10^–8^) for particularly the thrombus and fibrin parameters at later time points (Suppl. Fig. S4). For the TF microspots, correlations between parameters were substantially higher (Suppl. Fig. S4), thus pointing to a consistent, blood donor-dependent thrombus signature across the collagen-related microspots.

### TF-dependent regulation of intrinsic coagulation pathway

The findings so far pointed to a consistent accelerating role of the extrinsic TF/FVIIa pathway, mostly on parameters of thrombus and fibrin formation. As earlier flow studies suggested that the intrinsic FXII-triggered coagulation pathway is relatively slow in onset^13^, we also examined these kinetics. Therefore, citrate-anticoagulated blood samples were pre-incubated with blocking agents, inactive FVIIa (iFVIIa, 1 μM) and/or CTI (40 μg/mL, added upon blood drawing), and flowed over microspots *M1–4* and *M6–7* with(out) co-coated TF at high shear rate (1,000 s^-1^).

**Figure 3:**
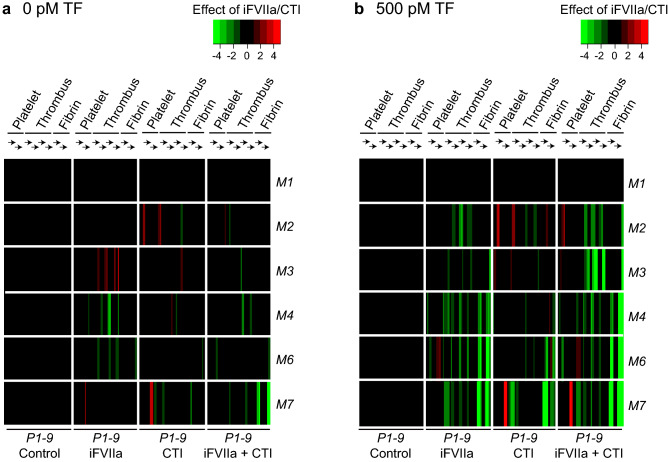
Additive contribution of extrinsic and intrinsic coagulation pathways to kinetics of whole blood thrombus formation. Blood from control subjects was collected into citrate with or without CTI (40 μg/mL); where indicated, samples were supplemented with iFVIIa (1 μM). Whole blood perfusion (1,000 s^-1^) was over microspots *M1–7* (± co-coated TF, 500 pM). Analysis of parameters *P1–9* over time ( →) was carried out, as for Fig. 2. Four conditions (± iFVIIa ± CTI) were compared per subject (*n* = 4–6). Data were scaled (0–10) per parameter (*P1–9*) across surfaces. (**A**) Subtraction heatmap representing relevant effects of CTI, iFVIIa or CTI + iFVIIa (no TF). (**B**) Idem for microspots with TF. Effects were filtered for changes outside the range of means ± SD. Data File 3 provides full data.

In the absence of TF, blocking the extrinsic pathway with iFVIIa decreased only the thrombus parameters on *M4,6* (Fig. 3A). As expected, iFVIIa caused a pronounced suppression of both thrombus and fibrin parameters on TF-containing microspots (Fig. 3B), confirming its TF-inactivating, thrombin-suppressing effect. On the other hand, CTI had a moderate inhibitory effect on fibrin formation in the absence of TF, but a substantially larger inhibitory effect with TF present, especially for the reactive (GPVI-dependent) surfaces *M6–7*. Strikingly, with TF present, the combination of iFVIIa and CTI had a stronger inhibitory effect on thrombus (*P3–6*) and fibrin (*P7–9*) parameters for surfaces *M2–4* than iFVIIa alone (Fig. 3B). Similarly, for microspots *M6–7* plus TF, the iFVIIa + CTI combination reduced fibrin formation more profoundly than iFVIIa or CTI did alone (medians, *p* = 0.001 for *M6*, *p* = 0.01 for *M7*). Accordingly, these results indicated a stimulating effect of immobilized TF on the intrinsic (FXII) coagulation pathway concerning overall thrombus formation and fibrin clot formation.

### Prediction modelling of extrinsic and intrinsic coagulation pathways

Using the raw datasets of all flow runs (Data File 2), we performed mathematical modelling to predict values for the separate or combined presence of TF, iFVIIa and/or CTI, also assuming interactions between the pathways (Suppl. Fig. S3). Using the constructed model, prediction for the presence of either TF, iFVIIa or CTI increased from *M2* (mean 54%) to *M7* (mean 74%), in agreement with an overall increase in effects of these interventions on the microspots (Suppl. Table S1). A similar increase was seen, when calculating the prediction accuracy of combined effects. This modelling hence indicated that the presence of TF, iFVIIa and/or CTI resulted in a consistent pattern of platelet–fibrin thrombus formation, especially on the active collagen and collagen-related microspots.

### Role of thrombomodulin-protein C anticoagulant pathway

**Figure 4:**
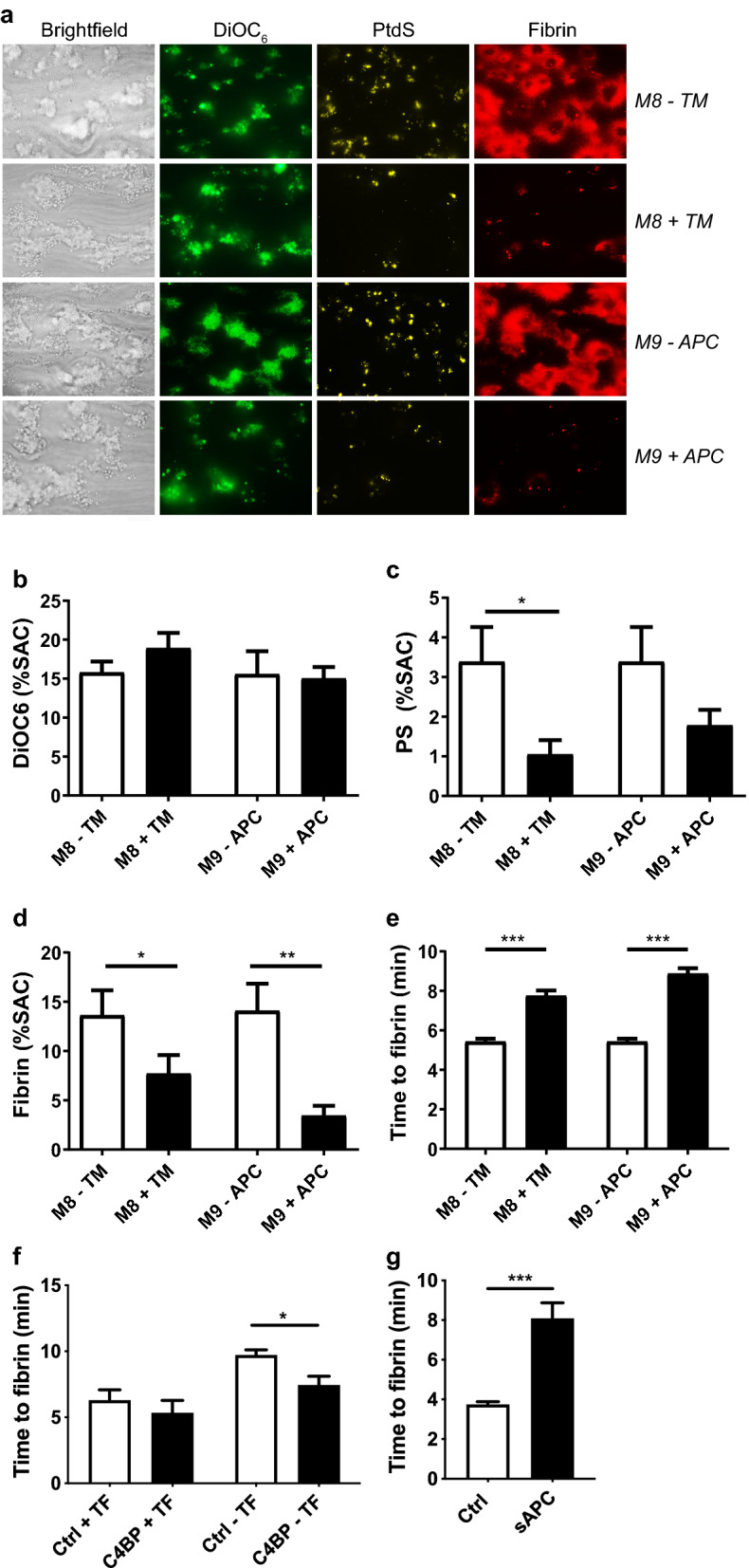
Alterations in whole blood thrombus formation by immobilized thrombomodulin or activated protein C (APC). (**A**–**E**) Collagen-I microspots were co-coated or not with TM (*M8*) or APC (*M9*), and subjected to whole-blood flow at 1,000 s^-1^ (TF in recalcification medium). (**A**) Representative bright-field and fluorescence microscopic images, as in Fig. 1, taken after 6 min; bars = 20 µm. (**B**–**D**) Platelet deposition (**B**), phosphatidylserine (**C**) and fibrin formation (**D**) after 6 min of flow. (**E**), Time to fibrin formation per microspotted surface, demonstrating delaying effects of TM and APC. Means ± SEM. *n* = 10–11, *p < 0.05. **p < 0.01, ***p < 0.001. (**F**,**G**) C4BP or soluble APC (sAPC) were added to whole blood before flow perfusion over collagen-I microspots co-coated or not with TF (*M6*). Time to fibrin formation (*n* = 4–7) in the presence of C4BP (**F**) or sAPC (**G**).

Endothelial thrombomodulin (TM) supports anticoagulation, by converting protein C into the anticoagulant protease APC in the presence of thrombin^9^. Once formed, APC operates in complex with protein S to cleave and inactivate FVa and FVIIIa. Since *M6* provided an active procoagulant surface (*P2*), we used these collagen-I microspots to assess possible anticoagulant effects of immobilized TM or APC in microspots. Extrinsic coagulation here was triggered by the addition of TF in recalcification medium. Microscopic imaging after 6 min indicated that co-coating of collagen-I with TM (*M8*) or APC (*M9*) left platelet adhesion unchanged, but markedly lowered platelet phosphatidylserine expression (Fig. 4A–C). In addition, the formation of fibrin was significantly impaired (*p* < 0.001) (Fig. 4D,E). Subtraction heatmaps also disclosed a major inhibition of the co-coating of TM or APC on fibrin parameters *P7–9* (Suppl. Fig. S5). In agreement with these findings, TM reduced thrombin generation profiles in plasmas from control subjects (Suppl. Fig. S5). This suggested that the neighbouring TM or APC diminished thrombin activity, likely by on-spot inactivation of coagulation factors.

In agreement with a role of the protein C pathway under flow, the addition of C4BP, known to bind and decrease protein S activity^20^, caused a significant decrease in time to fibrin on collagen-I microspots (*M6*) (Fig. 4F). Furthermore, the addition of soluble APC (sAPC) to the blood revealed a similar delay in fibrin formation as immobilized APC (Fig. 4G). Together, these data pointed to a regulatory role of the pathway of TM, APC and protein S in the later stage of flow-dependent platelet–fibrin thrombus formation.

### Altered thrombus formation by congenital coagulation factor abnormalities

To assess how the thrombus formation was altered in blood samples from patients with defects in coagulation or anticoagulation factors, we selected three sets of GPVI-activating microspots with a robust thrombus build-up, i.e. *M6* (collagen-I ± TF); *M7* (GFOGER-GPO ± TF); and *M8* with sensitivity for anticoagulation (collagen-I ± TM; TF in recalcification medium). Blood was obtained from eight representative patients with known factor abnormalities, associated with either a bleeding or a prothrombotic propensity (Table 2). Of note, one patient with FXI deficiency used aspirin medication. Ten healthy subjects served as day controls.

**Table 2:** Characteristics of patients with coagulation factor abnormalities and corresponding control subjects.

Subjects	Deficiency	Age (y)	Male/female	Platelets (10^9^/L)	Haematocrit (%)	Medication	Factor level	Refs.
Ctrl 1–10	none	30–51	5 M, 5F	214–248	40.2–45.4	None	Normal	n.a
Pat 1	FXII	60	M	306	48.8	Aspirin	< 1% FXII	n.a
Pat 2	FXII	44	F	198	33.3	None	< 1% FXII	n.a
Pat 3	FXI	18	M	260	44.1	None	19% FXI	n.a
Pat 4	FXI	31	F	292	40.2	None	33% FXI	n.a
Pat 5	FV	72	F	257	34.9	None	< 5% FV	^22^
Pat 6	FV	75	F	132	38.8	None	< 5% FV	^22^
Pat 7	FV-Leiden	48	F	257	38.6	None	*F5* 1691G → A	^45^
Pat 8	FV-Leiden	38	F	267	37.7	None	*F5* 1691G → A	^45^

^a^Confirmed mutation in SHBG (sex hormone binding globulin) region (unpublished).

**Figure 5:**
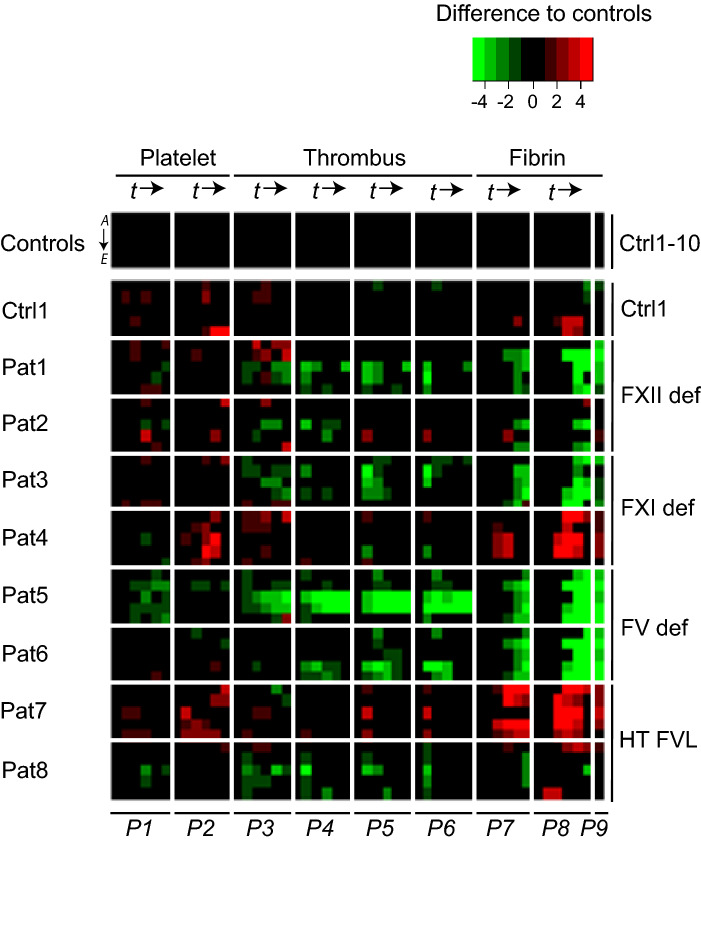
Altered thrombus and fibrin formation in patients with coagulation factor abnormalities. Blood samples from healthy day control subjects (*n* = 10) and patients (Pat, *n* = 8) with indicated coagulation factor abnormalities were investigated for platelet, thrombus, and fibrin formation at a shear rate of 1,000 s^-1^, with image capturing every 2 min (see Fig. 2). Image parameters (*P1–9*) were obtained for microspot *M6* no TF *(rows A)* or with TF *(rows B)*; *M7* no TF *(rows C)* or with TF *(rows D)*; *M8* with TF (*rows E*). Data were univariate scaled (0–10) across surfaces per parameter for all flow runs. Differential heatmap of scaled parameters over time per patient in comparison to means of control subjects. Filtering applied for changes outside the range of means ± SD of control subjects. A green colour indicates a relevant decrease, a red colour a relevant increase in comparison to controls. Full data are given in Data File 5.

For structured analysis, subtraction heatmaps were generated of scaled parameter values (Fig. 5), showing relevant changes in platelet (*P1–2*), thrombus (*P3–6*) and fibrin (*P7–9*) parameters. Subtraction heatmaps after 6 min of flow (Suppl. Fig. S6) were in line with the integrated results. Simultaneously, plasma samples collected from controls and patients were used for assessment of thrombin generation curves (Suppl. Fig. S6).

In comparison to the overall control samples, blood from patients 1–2, with a severe FXII deficiency (< 1%), was unchanged regarding platelet activation (*P1–2*), but overall reduced in parameters of thrombus and fibrin formation (*P3,7,8*), when TF was present (Fig. 5). This agrees with the indications for a positive interaction of the intrinsic and extrinsic pathways. Typically, patient 1 demonstrated a more extensive reduction than patient 2 in extent of thrombus and fibrin formation over time (*P3–9*), which may be related to the intake of aspirin. Ellagic acid-induced thrombin generation in plasma was more severely reduced for patient 1 than for patient 2 (Suppl. Fig. S6).

Across all microspots, a strong FXI deficiency (19%) in patient 3 was accompanied by a consistent impairment in (early) thrombus and (late) fibrin parameters (*P3–9*). On the other hand, blood from patient 4 with limited reduction in FXI (33%) showed a relative increase in parameters of platelet activation (*P2*) and fibrin formation (*P7–9*) (Fig. 5), for all microspots. Plasmas from patients 3 and 4 showed the expected impairments in patterns of thrombin generation (Suppl. Fig. S6).

Patients 5–6 were included because of severe congenital deficiency in FV (< 5%), associated with mild to severe bleeding symptoms^21, 22^. In the whole-blood flow assay, for all microspots, but most strongly for *M7*, fibrin parameters were consistently impaired (Fig. 5), which is also noticed in the 6-min heatmap (Suppl. Fig. S6). For both patients, the lower fibrin formation was preceded by a steady and profound reduction in thrombus parameters. Thrombin generation triggered by ellagic acid or TF was also greatly suppressed in the patients’ plasmas (Suppl. Fig. S6).

Blood was furthermore analysed for patients 7–8, with a heterozygous FV-Leiden mutation, i.e. a moderate prothrombotic propensity. For patient 7, we found an overall increased platelet activation (*P2*) and fibrin formation (*P7–9*). This was compatible with higher TF-triggered parameters of thrombin generation. Markedly, for patient 8 no such changes were seen in either test.

**Figure 6:**
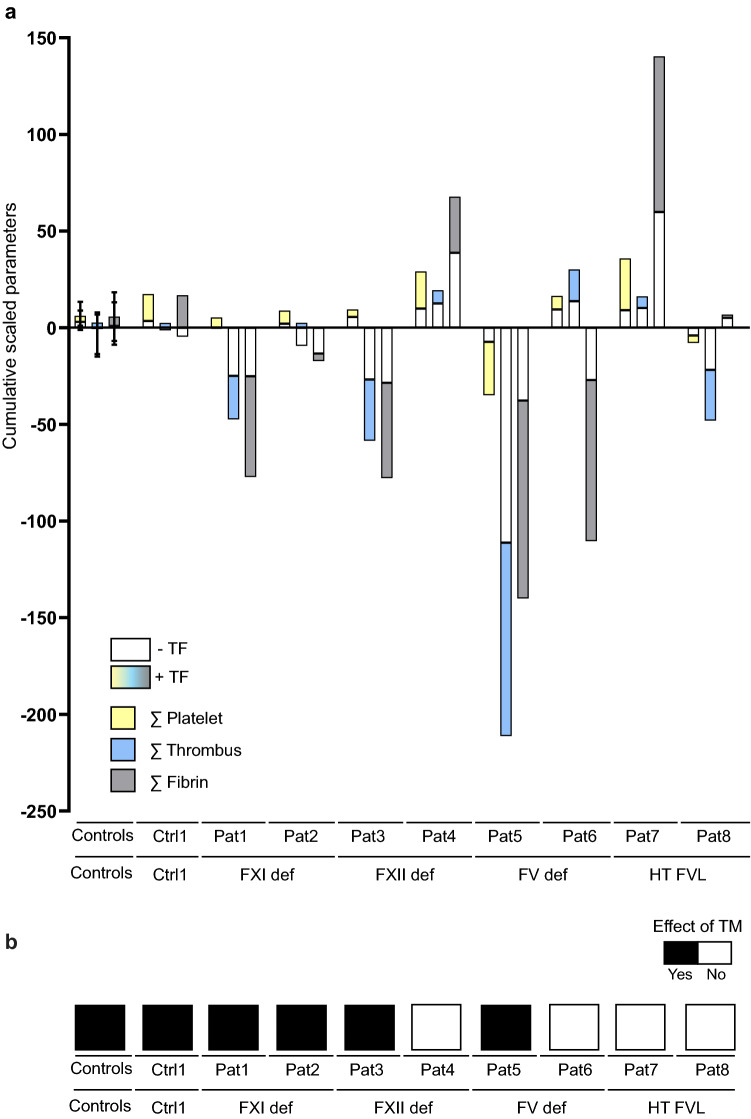
Integrated changes in thrombus formation in the presence or absence of TF or TM for individual patients with coagulation factor abnormalities. Microspot-based formation of platelet–fibrin thrombi was measured in whole-blood from 10 control subjects and 8 patients with coagulation factor abnormalities, as in Fig. 5. Relevant changes of scaled parameters in comparison to means of control subjects (Ctrl, outside range of means ± SD) were integrated for all time points. Bars show results from all controls (means ± SD), from a representative control (Ctr1) and all individual patients (Pat1 to Pat8). (**A**) Cumulative relevant changes of scaled parameters of platelet adhesion (*P1–2*), thrombus signature (*P3–6*) and fibrin formation (*P7–9*) across five surfaces. (**B**) Mean relevant effects of TM on fibrin parameters for control subjects and patients, assessed for *M8*.

As an integrative approach to compare the alterations in patient samples, we combined all relevant changes in platelet, thrombus and fibrin parameters to obtain a net summation of the decreases and increases in comparison to the samples from 10 healthy control subjects (Fig. 6A). This calculation confirmed the overall decreases in thrombus and fibrin parameters for blood from patients 1 (FXII), 3 (FXI) and 5–6 (FV). It furthermore showed increased platelet parameters in blood from patients 4 (FXI) and 7 (FV-Leiden), all showing a gain-of-function in fibrin-thrombus formation. Consistent with this, changes in the patients' blood samples were larger in the presence of TF (coloured bars of Fig. 6A) than in the absence of TF (white bars). This underlined that TF-containing microspots were more discriminative in revealing altered flow-dependent fibrin-thrombus formation than the non-TF microspots.

To evaluate the changes in TM-protein C pathway in the patient blood samples, the observed effects of TM as scaled fibrin parameters (*P7–9*) were integrated to obtain a mean effect parameter. In comparison to the overall positive TM effect in control samples (black bars in Fig. 6B), we noticed a consistent TM effect in blood from patients 1–3, but not for the 'reactive' blood from patient 4. Importantly, TM effects were abolished with blood from patients 7–8 with protein C pathway defects, i.e. FV-Leiden mutation.

## Discussion

In this study we have developed a multi-parameter microfluidics platform to determine the kinetics and interactions of thrombus formation in flowing whole blood under conditions that trigger specific platelet receptors and (anti)coagulation pathways. Key elements of the current optimized procedure are: (*1*) controlled two-step mixing of citrated blood with recalcification medium; (*2*) use of combined microspots with or without co-coating of (procoagulant) TF and/or (anticoagulant) TM or APC; (*3*) kinetic measurements of multiple processes from series of multicolour microscopic images; (*4*) standardized image analysis. As a result, 41 parameter values per microspot were obtained, providing simultaneous information on platelet adhesion/activation, thrombus signature, and fibrin formation at defined flow and shear rates. This multiparameter analyses revealed novel insights into: (*1*) the comparative and inter-dependency of the intrinsic and extrinsic ways of fibrin formation; (*2*) the identified local role of the protein C pathway in platelet-dependent fibrin formation; (*3*) the fact that similar congenital abnormalities in coagulation factors can result in distinct kinetics of clotting activity under flow.

This approach of systematically comparing time profiles of thrombus formation on multiple platelet adhesive surfaces provided new insights into the regulatory pathways of the clotting process under physiological as well as pathophysiological conditions. Regardless of the surface, we found an overall dose-dependent enhancing effect of TF on thrombus build-up and contraction, preceding the formation of a fibrin clot. This pointed to a priming effect of (thrombin-induced) platelet activation for the onset of clotting under flow. In addition, the enhancing effect of TF appeared to be independent of the shear rate (1,000 or 150 s^-1^), while it was less pronounced on highly platelet-reactive surfaces (causing strong GPVI activation). These data extend the earlier reported effect of coated TF in combination with collagen-I on fibrin formation^18,23^.

Interventions to trigger (TF) or block the extrinsic (iFVIIa) or intrinsic (CTI) coagulation pathways provided novel information on the interactions between the two pathways in flowing whole blood. Strikingly, for all microspot surfaces, we found larger effects of CTI on thrombus and fibrin parameters, when TF was present. Indeed, modelling of the predicted contribution of either pathway also revealed positive interactions of iFVIIa and CTI interventions, in particular for *M6,7*. Our findings hence point to a new mechanism of intrinsic coagulation activation under flow that is promoted by the extrinsic TF pathway.

Various mechanisms can be responsible for this coagulation crosstalk. Activated platelets are known to secrete polyphosphate particles, which trigger FXII activation^14^. Furthermore, studies with knock-out mice revealed a role of FXI and FXII in collagen-dependent thrombus formation in vivo and ex vivo^13,24,25^. The same studies also demonstrated a role of TF, later stabilized by FXII in vivo, hence pointing towards a TF-priming effect of FXII activation. Interestingly, these findings confirm very early research papers describing a role for platelets in the contact phase of blood coagulation^26^.

Strikingly, the present microfluidics technique also allowed detection of the natural anticoagulant protein C pathway, involving TM and APC. Herein, TM acts as a cofactor for the activation of protein C that together with protein S inactivates FVa and FVIIIa, thus abrogating the formation of thrombin and fibrin^27^. Other authors have already established that, in a static plasma system, the addition of TM or APC suppresses thrombin generation, which effect is partly impaired in plasmas from carriers of the FV Leiden mutation^28–30^. Our group has shown that in flow adhesion the phosphatidylserine-exposing platelets can act as accumulating sites for FVa and FVIIIa^19^. Together, this points to the presence of a local and acute mechanism of TM-dependent protein C activation, causing inactivation of FVa and FVIIIa, which operates on microspots in flowing whole blood. The existence of a pathway of prothrombin activation by APC in flowing plasma was reported before^31,32^.

To determine the pathophysiological implications of these findings, we examined blood samples from 8 patients with (anti)coagulation defects. Overall, the alterations in flow-dependent clot formation were in agreement with the clinical phenotypes. For instance, in case of particular coagulation factor deficiencies (FV, FXI, FXII) a decrease in thrombin and fibrin parameters, versus an increase in parameters in patients with procoagulant alterations (FV-Leiden). Markedly, platelet parameters were only decreased in case of severe FV-deficiency, followed by impairments in thrombus and fibrin parameters. On the other hand, we noted heterogeneity between patients with the same factor abnormality, although this is in general correlated with differences in thrombin generation profiles. The reason for this is unclear, but it has been shown that changes in a number of coagulation factor levels can influence the extent of APC resistance, such as detected in specialized thrombin generation tests, e.g. in plasmas from FV-Leiden patients^33–35^. Clearly, the present whole-blood technique has as advantage that it integrates the alterations in platelet and plasmatic (anti)coagulation factors. Analysis of the patient samples further confirmed the priming effect of platelet adhesion and activation for the clotting process.

Taken together, our findings indicate that the approach of combined phenotyping of platelet–fibrin thrombus formation in whole blood can serve as the basis of a novel, integrative way of determining: (*1*) interaction mechanisms of the platelet and (anti)coagulation pathways, (*2*) haemostatic traits due to genetic or acquired aberrations in coagulation factors, and (*3*) multi-parameter algorithms for assessment of (combined) platelet and coagulation defects. These microfluidic methods can further aid as an important functional add-on to genetic information coming from full or partial genomic sequencing protocols.

## Methods

### Materials

Collagen type I (Horm) was from Takeda (Hoofddorp, The Netherlands). Collagen type III (C4407), fibrinogen (F3879), and laminin 511/521 (L6274) were from Sigma-Aldrich (Zwijndrecht, The Netherlands). VWF from human plasma was isolated to homogeneity, as described^36^. Recombinant human TF was from Dade-Behring (Breda, The Netherlands); thrombomodulin (TM) from American Diagnostica (Pfungstadt, Germany); activated protein C (APC) from Innovative Research (Novi, MI, USA). Rhodocytin was purified from the venom of the Malayan pit viper, *Calloselasma rhodostoma*^37^ and was a generous gift of Dr. K. Clemetson (Bern University, Switzerland)*.* C4BP was obtained from Athens Research (Athens, GA, USA). Chemically synthesized triple-helical peptides were obtained from the Dept. Biochemistry, University of Cambridge (United Kingdom), and used as described^10,36^: H-GPC(GPO)_3_GFOGER(GPO)_3_GPC-NH_2_ (GFOGER-GPO); and VWF-binding peptide H-GPC(GPP)_5_GPRGQOGVMGFO(GPP)_5_GPC-NH_2_ (VWF-BP). Bovine serum albumin (BSA) was obtained from Sigma-Aldrich; d-phenylalanyl-l-prolyl-l-arginine chloromethyl ketone (PPACK) from Calbiochem (San Diego, CA, USA); corn trypsin inhibitor (CTI) from Haematologic Technologies (Essex Junction, VT, USA), and Gly-Pro-Arg-Pro from Stago (Parsippany, JN, USA). Fragmin was obtained from Pfizer (Capelle a/d IJssel, The Netherlands), inactivated factor VIIa (iFVIIa) from NovoNordisk^38^ (Bagsvaerd, Denmark). 3,3′dihexyloxa carbocyanine iodide (DiOC_6_) from AnaSpec (Fremont, CA, USA); Alexa Fluor (AF)568-labeled annexin A5 from Life Technology (Carlsbad, CA, USA); and AF647-labeled human fibrinogen from Molecular Probes (Eugene, OR, USA).

### Blood donors and blood collection

Blood was obtained from healthy volunteers free from antiplatelet or anticoagulant medication for at least 4 weeks. Patients and corresponding control subjects were obtained from the screening program for familial plasma-based causes of bleeding or thrombophilia at the University Hospital of Padua (Italy)^39^. Patient characteristics and medication use are reported in Table 2. Studies were approved by the local Medical Ethics Committees (Maastricht University Medical Centre, NL31480.068.10). All subjects gave full informed consent according to the Declaration of Helsinki and all methods were performed in accordance with the relevant guidelines and regulations.

Blood was drawn by venepuncture using a vacuum container, and collected into 9 mL tubes containing 3.2% trisodium citrate (Greiner, Alphen a/d Rijn, The Netherlands). Blood cell counts and other haematological parameters were assessed with a Sysmex XN-9000 analyser (Sysmex, Chuo-ku, Kobe, Japan). The values were within the normal ranges, except where indicated otherwise. Global coagulation times were only assessed, if aberrant thrombin or fibrin generation was suspected or detected^40^. For microfluidics studies, blood samples were kept at room temperature and used within 4 h. Blood quality was checked during measurements. Where indicated, blood samples were centrifuged twice to obtain platelet-free plasmas^41^. The plasmas were snap-frozen, and stored at − 80 °C until use.

### Preparation of microspot coatings

Using a precision mall, cleaned and degreased glass coverslips (24 × 60 mm, Thermo-Fisher, Breda, The Netherlands) were fresh daily coated with two microspots (~ 1 mm diameter, 5 mm centre-to-centre distance), with or without TF, TM or APC. To prevent cross-over effects, the most active microspot (with TF or without TM/APC) was placed downstream^11^. Coating was performed with 2.0 μL of the following solutions (Table 1): *M1,* BSA control (1% BSA-containing blocking buffer); *M2,* rhodocytin (250 μg/mL) + VWF (50 μg/mL); *M3,* laminin (100 μg/mL) + VWF (50 μg/mL); *M4,* collagen-III (100 μg/mL); *M5,* collagen-I low (10 μg/mL); *M6, M8–9*, collagen-I high (50 μg/mL); and *M7,* GFOGER-GPO (250 μg/mL) + VWF-BP (100 μg/mL). Coating concentrations were optimized to achieve maximal platelet adhesion, as described^10^. Where indicated, suspensions of TF, TM or APC (2.0 μL) were post-coated after 1 h at one of the spots (after washing with saline). For *M1–7*, maximal co-coating concentrations of TF (500 pM); for *M8*, TM (10 nM or 0.7 μg/mL); for *M9*, APC (10 nM or 0.6 μg/mL). Before start of the regular measurements, the activity of co-coated TF was checked as a consistent acceleration of fibrin formation.

Absence of cross-over effects between the two microspots was verified by altering positions and checking key parameters of thrombus formation^11^. After coating, the coverslips were blocked with BSA-containing Hepes buffer pH 7.45 (136 mM NaCl, 10 mM Hepes, 2.7 mM KCl, 2 mM MgCl_2_, 0.1% glucose, 1% BSA), mounted onto a transparent flow chamber (height 50 μm, width 3.0 mm, length 30 mm), and pre-rinsed with Hepes buffer pH 7.45 containing 0.1% BSA^10^.

### Recalcification of blood and mixing in the flow chamber

During recalcification, samples of citrated blood (1,000 µL) were perfused through a flow chamber using two pulse-free micro-pumps (Model 11 Plus, 70–2,212, Harvard Apparatus, Holliston, MA, USA), and a *y-*shaped mixing tubing. The recalcification medium for co-perfusion consisted of 32 mM MgCl_2_ and 63 mM CaCl_2_ in Hepes buffer pH 7.45 (in another 1 mL syringe). Mixing was at a volume ratio of 10 (blood) to 1 (recalcification medium), at flow rates to achieve the calculated wall shear rate in the chamber of 150 or 1,000 s^-1^. Additional mixing of the blood and medium was achieved by sudden flow profile change from round to flat at the flow-chamber entry side; complete mixing was confirmed by computer modelling of the fluid dynamics (Suppl. Fig. S1). Where indicated, samples were pre-incubated with FVIIa inhibitor (iFVIIa, 1.0 μM) and/or corn trypsin inhibitor (CTI, 40 μg/mL), which was added during blood taking. Typically, for experiments with microspots *M8–9*, TF (10 pM) was added to the recalcification medium. As a standard procedure, blood samples were pre-labelled with final concentrations of DiOC_6_, (0.5 μg/mL, staining all platelets), AF647-fibrinogen (8.5 μg/mL), and AF568-annexin A5 (4.0 μg/mL, staining phosphatidylserine-exposing platelets).

Full mixing of blood with recalcification medium was achieved by a simple, but effective two-step mixing procedure. The first step was provided by an in house fabricated *y*-shaped Versitec silicone tubing (1.0 mm ID, 3.0 mm OD, Saint-Gobain Plastics, France); this was constructed by perforating the tubing laterally (at 1 cm from outlet, angle 30°) with a 18G needle placed inside-out, after which a second tubing was connected to the needle tip. Leakage-free sealing was obtained using liquid polymerizing silicone; the needle was removed after polymerization. This resulted in a small pore (~ 0.5 mm) in the main tubing wall, through which the recalcification medium could be pressure-injected. The second mixing step was provided at the flow chamber inlet, by transition from a tubular (1 mm Ø) to flat (50 µm height, 3 mm width) cross-section, resulting in sudden redistribution of the flow velocity profile. This set up ensured consistent mixing, a low shear rate at the inlet, and a higher-shear laminar flow pattern inside the parallel-plate chamber (Suppl. Fig. S1).

### Real-time detection of platelet–fibrin thrombus formation on microspots

Bright-field and fluorescence microscopic images were acquired from each of the microspots during blood flow at t = 0, 2, 4, 6, 8 (to 10) min. Image recording was performed with an inverted EVOS fluorescence microscope (Life Technology, Ledeberg, Belgium), equipped with bright-field illumination, three LED diode cubes (GFP 470 nm, RFP 531 nm and Cy5 626 nm), and an Olympus 60** × **oil-immersion objective with high *z*-axis resolution (UPLSAPO60, numerical aperture 1.35). Monochromatic images were collected at 8 bit (1,360 × 1,024 pixels, resolution 0.108 μm per pixel). In specific cases, images were collected using a multicolour confocal microscope (similar excitation wavelengths), as described^11^. Duplicate flow runs were performed per blood sample, or triplicate runs in case of marked variation.

### Standardized image analysis and delineation of thrombus outcome parameters

Images were analysed using semi-automated scripts, written in the open-access program Fiji^42^. Specific scripts were designed for images of bright-field and each fluorescent label. In the scripts, optimized fast Fourier transformation was applied to reduce background noise and filtering down image-wide structures. Subsequently, morphological vertical and horizontal dilate and erode steps were applied to remove noise pixels and to enhance relevant structures. Overlay images were generated to verify the binary mask images, and scripts looped back to reset thresholds if the analysis was incorrect. Thresholding was checked against t = 0 images.

Together, the binary bright-field and fluorescence images gave nine parameters (*P1–9*) per time point (see Table 1). The DiOC_6_ images reported on platelet deposition (*P1*); while the AF568-annexin A5 images indicated phosphatidylserine-exposing platelets (*P2*). Enhanced bright-field images were also analysed for platelet adhesion (*P3*). Bright-field images were analysed for thrombus morphology (*P4*): 0, no or few adhered platelets; 1, multiple single adhered platelets; 2, extensive coverage of single adhered platelets; 3, small platelet aggregates; 4, medium-size aggregates and thrombi; 5, large aggregates and thrombi^10^. Bright-field images were also scored (scale 0–3) for thrombus aggregation (*P5*) and thrombus contraction (*P6*). Regarding fibrin, AF647-fibrin(ogen) images provided information on fibrin deposition (*P7*), with thresholds set above values for fibrinogen binding^11^; bright-field images further gave a fibrin score on a scale of 0–3 (*P8*). Furthermore, shortening of the time to first fibrin formation (default *t* = 11 min) were recorded (*P9*). Fibrin formation was precisely quantified in real time from the appearance of growing fibres protruding from the platelet thrombi^18^. All scoring was in comparison to a set of pre-selected, representative images. Analysts were blinded to the experimental conditions. As described before, variation of key thrombus forming parameters for collagen-I was 15% (median)^43^. In addition, intra-individual variation of time to fibrin was 7–12%.

### Calibrated automated thrombin generation

Thrombin generation was measured in platelet-free plasma samples (total volume 120 μL) using a 96 wells plate assay, basically as described^41^. Briefly, 80 μL plasma samples were incubated with 4 μM phospholipid vesicles (PS/PE/PC, 20/20/60, w/w/w) at 37 °C for 10 min, and subsequently mixed with 20 μL prewarmed trigger reagent containing either TF (1 pM, f.c.), ellagic acid (10 μg/mL, f.c.) or TM (0.325 nM, f.c.) and with 20 μL fluorogenic thrombin substrate (Z-Gly-Gly-Arg AMC) containing CaCl_2_. After mixing, thrombin generation was measured per well using Thrombinoscope software (Thrombinoscope, Maastricht, The Netherlands). Standard curve parameters were obtained, as indicated in Table 1.

### Predictions of contribution of extrinsic and intrinsic pathways of coagulation

Models were made in Matlab for the predicted contribution of parameters to thrombus formation at all time points, using the conditions with or without TF, CTI or iFVIIa, thus making in total 8 combinations or classes. Raw data were loaded into Matlab, and (few) missing values were estimated using the function Knnimpute. Per microspot, 8-class predictions were run, in each case using 17 different machine learning programs (Weka classifiers: SimpleCart, BayesNet, NaiveBayer, Logistic, RBFNetwork, SMO, AggregationPerception, IB1, ConjunctiveRule, JRip, NNge, PART, BFTree, FT, J48, RandomForest, REPTree). Accuracies were calculated by leave-one-out cross validation. The predictions tested the hypothesis if the presence/absence of TF, iFVIIa or CTI significantly altered parameters of fibrin-thrombus formation per microspot. In other words, if the measured parameters allow to predict which of these factors is present. In addition, per class variable (TF, iFVIIa, CTI), t-tests (2-sided) were run to compare the performances of the 17 algorithms on every pair of surfaces, after correction for false discovery rate. These models were also checked by cross-validating, leave-one-out predictions. Linear regression models to predict *P3–9* outcomes over time from *P1–2* were also constructed in Matlab.

### Bioinformatics and statistics

Relationships between parameters were calculated by multiple regression analysis using the statistical package for social sciences (SPSS version 22). For comparative data analysis in heatmaps, mean values per parameter across surfaces were linearly scaled to a range from 0–10. One-way unsupervised hierarchical clustering was performed using the R version 3.2.5 (www.r-project.org). Euclidean distances were calculated, and clustering was by complete linkages. In order to visualize effects of TF co-coating or of interventions, scaled parameters were linearly subtracted to obtain subtraction heatmaps.

Numerical data of interventions are presented as means with SEM. Data from individual patients were compared, as described before^44^, to normal ranges established for a cohort of control subjects (set as means ± SD). Blood samples from patients and controls were analysed in parallel over the same period. Statistical comparison was by probability analysis (Mann–Whitey U-test for numerical or continuous variables). Values of *p* < 0.05 were considered significant.

## Supplementary information

Supplementary information

## References

[1] Heemskerk JW, Mattheij N, Cosemans JM (2013). Platelet-based coagulation: different populations, different functions. J. Thromb. Haemost..

[2] Alshehri OM (2015). Fibrin activates GPVI in human and mouse platelets. Blood.

[3] Mammadova-Bach E (2015). Platelet glycoprotein VI binds to polymerized fibrin and promotes thrombin generation. Blood.

[4] Podoplelova NA (2016). Coagulation factors bound to procoagulant platelets concentrate in cap structures to promote clotting. Blood.

[5] Mackman N, Tilley RE, Key NS (2007). Role of the extrinsic pathway of blood coagulation in hemostasis and thrombosis. Arterioscler. Thromb. Vasc. Biol..

[6] Jackson SP (2011). Arterial thrombosis: insidious, unpredictable and deadly. Nat. Med..

[7] Van der Meijden PEJ, Heemskerk JWM (2019). Platelet biology and functions: new concepts and future clinical perspectives *Nat*. Rev. Cardiol..

[8] Stalker TJ (2014). A systems approach to hemostasis: 3. Thrombus consolidation regulates intrathrombus solute transport and local thrombin activity. Blood.

[9] Versteeg HH, Heemskerk JW, Levi M, Reitsma PS (2013). New fundamentals in hemostasis. Physiol. Rev..

[10] De Witt SM (2014). Identification of platelet function defects by multi-parameter assessment of thrombus formation. Nat Commun..

[11] Swieringa F (2016). Platelet-dependent control of fibrin distribution and micro-elasticity in thrombus formation under flow. Arterioscler. Thromb. Vasc. Biol..

[12] Zhu S, Lu Y, Sinno T, Diamond SL (2016). Dynamics of thrombin generation and flux from clots during whole human blood flow over collagen/tissue factor surfaces. J. Biol. Chem..

[13] Van der Meijden PE (2009). Dual role of collagen in factor XII-dependent thrombus and clot formation. Blood.

[14] Verhoef JJ (2017). Polyphosphate nanoparticles on the platelet surface trigger contact system activation. Blood.

[15] Monroe DM, Hoffman M (2006). What does it take to make the perfect clot?. Arterioscler. Thromb. Vasc. Biol..

[16] Carnemolla R (2012). Quantitative analysis of thrombomodulin-mediated conversion of protein C to APC: translation from in vitro to in vivo. J. Immunol. Methods.

[17] Colace TV, Muthard RW, Diamond SL (2012). Thrombus growth and embolism on tissue factor-bearing collagen surfaces under flow: role of thrombin with and without fibrin. Arterioscler. Thromb. Vasc. Biol..

[18] Thomassen S (2018). Suppressive role of tissue factor pathway inhibitor-α in platelet-dependent fibrin formation under flow is restricted to low procoagulant strength. Thromb. Haemost..

[19] Swieringa F, Kuijpers MJ, Lamers MM, van der Meijden PE, Heemskerk JW (2015). Rate-limiting roles of the tenase complex of factors VIII and IX in platelet procoagulant activity and formation of platelet-fibrin thrombi under flow. Haematologica.

[20] Dahlbäck B (2011). C4b-binding protein: a forgotten factor in thrombosis and hemostasis. Semin. Thromb. Hemost..

[21] Duckers C (2008). Low plasma levels of tissue factor pathway inhibitor in patients with congenital factor V deficiency. Blood.

[22] Duckers C (2010). Residual platelet factor V ensures thrombin generation in patients with severe congenital factor V deficiency and mild bleeding symptoms. Blood.

[23] Okorie UM, Denney WS, Chatterjee MS, Neeves KB, Diamond SL (2008). Determination of surface tissue factor thresholds that trigger coagulation at venous and arterial shear rates: amplification of 100 fM circulating tissue factor requires flow. Blood.

[24] Kuijpers MJ (2014). Factor XIIa regulates the pathological process of thrombus formation on ruptured plaques. Arterioscler. Thromb. Vasc. Biol..

[25] Zilberman-Rudenko J (2016). Coagulation factor XI promotes distal platelet activation and single platelet consumption in the bloodstream under shear flow. Arterioscler. Thromb. Vasc. Biol..

[26] Walsh PN (1972). The role of platelets in the contact phase of blood coagulation. Br. J. Haematol..

[27] Dahlback B, Villoutreix BO (2005). The anticoagulant protein C pathway. FEBS Lett..

[28] Van 't Veer, C., Kalafatis, M., Bertina, R. M., Simioni, P. & Mann, K. G. Increased tissue factor-initiated prothrombin activation as a result of the Arg506t → Gln mutation in factor VLEIDEN. *J. Biol. Chem.* **272**, 20721–20729 (1997). 10.1074/jbc.272.33.20721 9252393

[29] Butenas, S., van 't Veer, C. & Mann, K. G. "Normal" thrombin generation. *Blood* **94**, 2169–2178 (1999). 10498586

[30] Curvers J (2002). Effects of hereditary and acquired risk factors of venous thrombosis on a thrombin generation-based APC resistance test. Thromb. Haemost..

[31] Van 't Veer, C., Hackeng, T. M., Biesbroeck, D., Sixma, J. J. & Bouma, B. N. Increased prothrombin activation in protein S-deficient plasma under flow conditions on endothelial cell matrix: an independent anticoagulant function of protein S in plasma. *Blood* **85**, 1815–1821 (1995). 7703488

[32] Lozano M (1996). Activated protein C inhibits thrombus formation in a system with flowing blood. Br. J. Haematol..

[33] Brugge JM (2005). Expression of the normal factor V allele modulates the APC resistance phenotype in heterozygous carriers of the factor V Leiden mutation. J. Thromb. Haemost..

[34] Castoldi E, Rosing J (2010). APC resistance: biological basis and acquired influences. J. Thromb. Haemost..

[35] Segers O, Simioni P, Tormene D, Castoldi E (2014). Influence of single nucleotide polymorphisms on thrombin generation in factor V Leiden heterozygotes. Thromb Haemost.

[36] Pugh N (2010). Synergism between platelet collagen receptors defined using receptor-specific collagen-mimetic peptide substrata in flowing blood. Blood.

[37] Hooley E (2008). The crystal structure of the platelet activator aggretin reveals a novel (ab)2 dimeric structure. Biochemistry.

[38] Sorensen BB (1997). Incorporation of an active site inhibitor in factor VIIa alters the affinity for tissue factor. J. Biol. Chem..

[39] Spiezia L (2018). ABO blood group and the risk of post-thrombotic syndrome. Ann. Hematol..

[40] Lancé MD (2012). Perioperative dilutional coagulopathy treated with fresh frozen plasma and fibrinogen concentrate: a prospective randomized intervention trial. Vox Sang..

[41] Loeffen R (2012). Preanalytic variables of thrombin generation: towards a standard procedure and validation of the method. J. Thromb. Haemost..

[42] Schindelin J (2012). Fiji: an open-source platform for biological-image analysis. Nat. Methods.

[43] Van Geffen JP (2019). High-throughput elucidation of thrombus formation reveals sources of platelet function variability. Haematologica.

[44] Nagy M (2018). Variable impairment of platelet functions in patients with severe, genetically linked immune deficiencies. Haematologica.

[45] Bertina RM (1994). Mutation in blood coagulation factor V associated with resistance to activated protein C. Nature.

