# Supplementary material for: Platelet-primed interactions of coagulation and anticoagulation pathways in flow-dependent thrombus formation
Source: Sci Rep. 2020 Jul 17;10:11910. doi: 10.1038/s41598-020-68438-9 (PMC7368055; doi:10.1038/s41598-020-68438-9)
Supplement: Supplementary file 1 — Supplementary information [file 41598_2020_68438_MOESM1_ESM.pdf]

Platelet-primed interactions of coagulation and anticoagulation pathways in flow-dependent thrombus formation

Sanne L. N. Brouns[1]*, Johanna P. van Geffen[1]*, Elena Campello[2]**, Frauke Swieringa[1,3]**, Luca Spiezia[2], René van Oerle[1], Isabella Provenzale[1], Remco Verdoold[1], Richard W. Farndale[4], Kenneth J. Clemetson[5], Henri M. H. Spronk[1], Paola E. J. van der Meijden[1], Rachel Cavill[6], Marijke J. E. Kuijpers[1], Elisabetta Castoldi[1], Paolo Simioni[2]*, Johan W. M. Heemskerk[1]*

* Equal contribution

[1]Departments of Biochemistry and Internal Medicine, Cardiovascular Research Institute Maastricht (CARIM), Maastricht University Medical Centre+, Maastricht, The Netherlands
[2]Department of Medicine, University of Padua Medical School, Padova, Italy
[3]Department of Protein Dynamics, Leibniz Institute for Analytical Sciences, ISAS, Dortmund, Germany
[4]Department of Biochemistry, University of Cambridge, Cambridge, UK
[5]Department of Haematology, Inselspital, University of Berne, Berne, Switzerland
[6]Department of Data Science and Knowledge Engineering, Maastricht University, Maastricht, The Netherlands

Supplemental files

Suppl. Table 1. Average performance of 17 algorithms in single class predictions. Predictions are expressed as percentage correct, in predicting thrombus formation parameters in the presence of TF, iFVIIa and/or CTI for microspots *M1-7*. Combined results of seven class predictions also indicated. Dark green colour points to high predictive performance.

%	TF	iFVIIa	CTI	Mean	Combined
M1	9.3	9.3	9.3	9.3	2.3
M2	42.1	55.5	64.2	53.9	12.3
M3	70.2	55.8	53.4	59.8	17.5
M4	87.6	62.4	61.0	70.3	24.2
M6	90.7	59.3	52.9	67.6	31.2
M7	81.2	67.5	73.5	74.1	38.4

Suppl. Table 2. Summed scaled effects of TF co-coating. Parameters *P1-9* were summed, net effect of TF was calculated and filtered for mean ± SD, as for Figure 2. Dark blue colours indicate higher summed effects.

Shear rate	Surface	Summed scaled effect of TF (P1-9)
$1000\ s^{-1}$	M1 (BSA)	0.00
	M2 (rhodocytin)	43.77
	M3 (laminin)	84.03
	M4 (collagen III)	36.20
	M5 (collagen I 20)	94.14
	M6 (collagen I 100)	65.29
	M7 (GFOGER-GPO)	6.55
	Average	*47.14*
$150\ s^{-1}$	M1 (BSA)	0.00
	M2 (rhodocytin)	26.42
	M3 (laminin)	13.71
	M4 (collagen III)	14.24
	M5 (collagen I 20)	103.79
	M6 (collagen I 100)	47.43
	M7 (GFOGER-GPO)	32.29
	Average	*33.98*

Supplemental figures

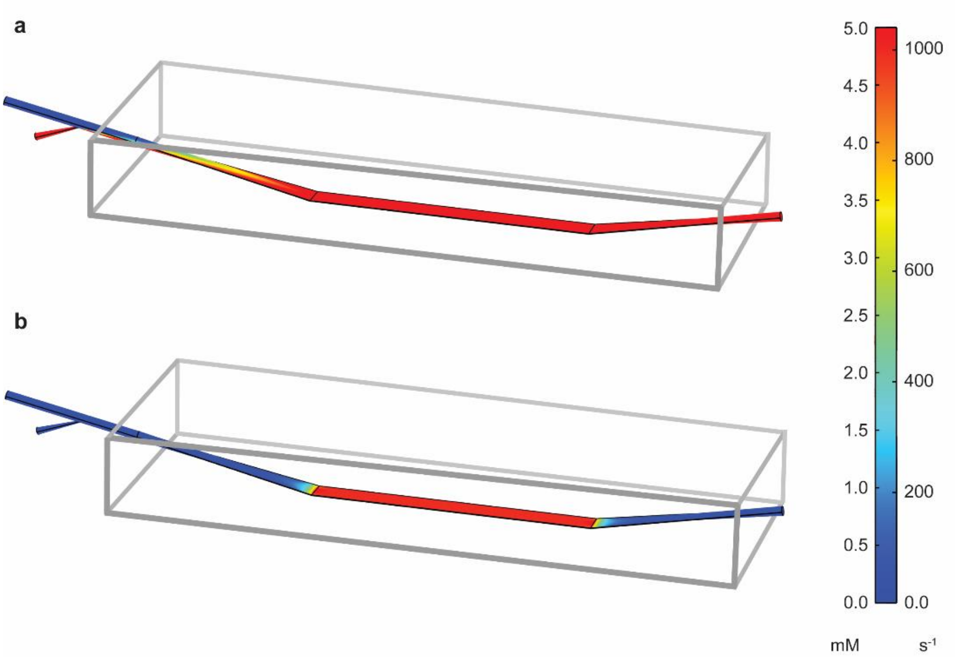

Suppl. Figure 1. Computational fluid dynamics modelling of mixing efficacy and shear rate. The three-dimensional structure of the microfluidics flow chamber (height 50 µm, width 3.0 mm, length 30 mm, 12° circular inlets and outlets) was digitized using Autodesk Inventor v2016 (San Rafael, CA, USA). The digital image was loaded into the computational fluid dynamics (CFD) package v4.2 of Comsol. Modelling was performed for whole-blood perfusion in the upper inlet at a rate of 4.5 mL/hour using a density of 1060 kg/m^3, a dynamic viscosity of 0.004 Pa•s, and a free Ca^{2+} concentration in citrated blood of 50 µM. Injection of a re-calcification medium (63.2 mM $CaCl_2$) in the *y*-shaped inlet was set at 0.45 mL/hour (no viscous stress at outlet). A physics-controlled mesh was used for laminar flow simulation, with a normal element size, resulting in 3.8 x 10^6 elements. Laminar flow was simulated using the module 'Direct solver', and the results were fed into the module 'Transport of diluted species' (equating for convection, diffusion and temperature), which thus simulated the extent of mixing of Ca^{2+} in the *y*-shaped inlet structure. Colour bars represent the calculated wall shear rate and the redistributed Ca^{2+} concentration. (A) *Y*-shaped dual inlet of recalcification medium injected into the blood with sudden fluid profile conversion from round to flat, giving complete fluid mixing. Coloured profile indicates mixing efficacy as calculated redistribution of free Ca^{2+}. (B) Coloured profile of wall shear rate post-mixing in microfluidic chamber.

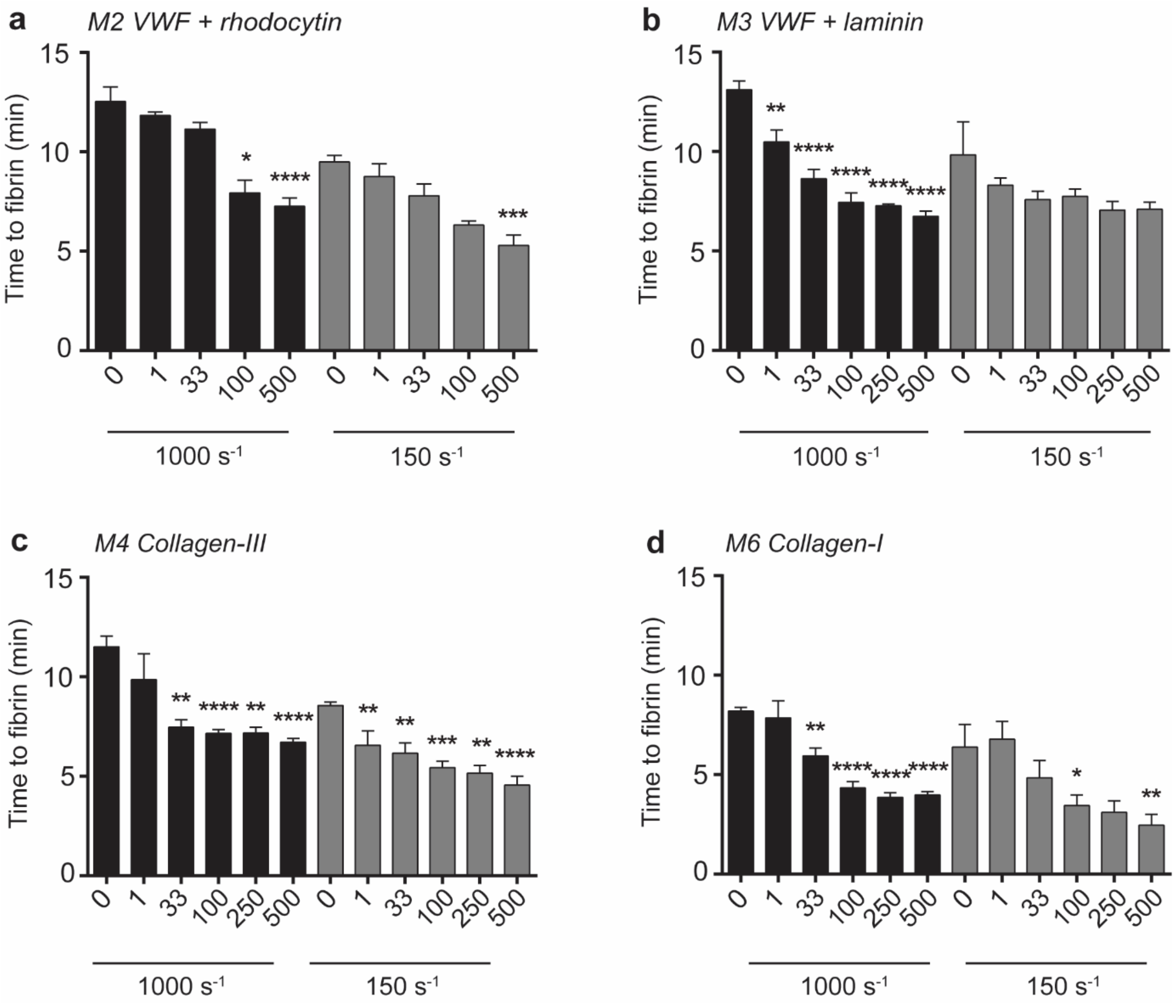

Suppl. Figure 2. Effect of tissue factor co-coating on fibrin formation times on different surfaces. Co-coating of microspots *M2-4* and *M6* with indicated concentrations of TF (0-500 pM TF). Whole-blood was perfused at a wall shear rate of 1000 s^{-1} or 150 s^{-1} as indicated for Figure 1. Indicated are times to first fibrin formation on VWF + rhodocytin (A), VWF + laminin (B), collagen-III (C) and collagen-I (D), obtained from differential images. Means ± SEM, *n* = 5-10, *p<0.05, **p<0.01, ***p<0.001 or ****p<0.0001.

a

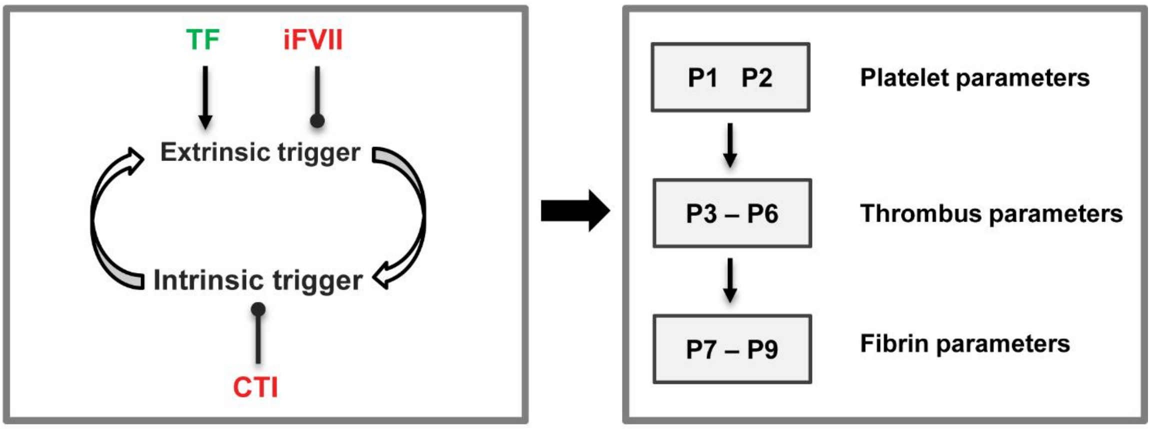

b

	- TF				+ TF			
	Thrombus		Fibrin		Thrombus		Fibrin	
	P1	*P2*	*P1*	*P2*	*P1*	*P2*	*P1*	*P2*
M2	3	3	0	1	3	3	0	0
M3	2	1	0	1	2	1	0	1
M4	2	0	0	1	4	1	1	0
M6	2	2	0	1	4	0	0	3
M7	4	3	2	0	4	3	0	0
Sum	13	9	2	4	17	8	1	4
Max.	20	20	15	15	20	20	15	16
Predicted %	65%	45%	13%	27%	85%	40%	7%	25%

Suppl. Figure 3. Prediction modelling of intrinsic and extrinsic pathway modulation on parameters of thrombus formation. (A) Schematic presentation of coagulation processes triggered via intrinsic (TF) and extrinsic (FXII) pathways to enhance consecutive sets of parameters of thrombus formation. Modulating effects of TF, iFVIIa and CTI on the basal model are indicated. (B) Linear regression models were constructed of univariate scaled parameters, using *P1* and *P2* to predict the accuracies of *P3-9*. The mathematical equation of this model was as follows: $P_i(t) = C_i + a_i P1(t) + b_i P2(t) + c_i t + d_i P1(t\text{-}1) + e_i P2(t\text{-}1)$, $3 \le i \le 9$. Assessed was per microspot surface (*M2-7*) the number of variables (*a-e*), using values of *P1*

and *P2* as an input, that contributed to a right prediction of thrombus parameters (*P3-6*) and fibrin (*P7-9*) parameters, in the absence or presence of TF. Tabled are percentages (%) of contributing variables, based on the maximums of 20 for thrombus parameters and 15 for fibrin parameters.

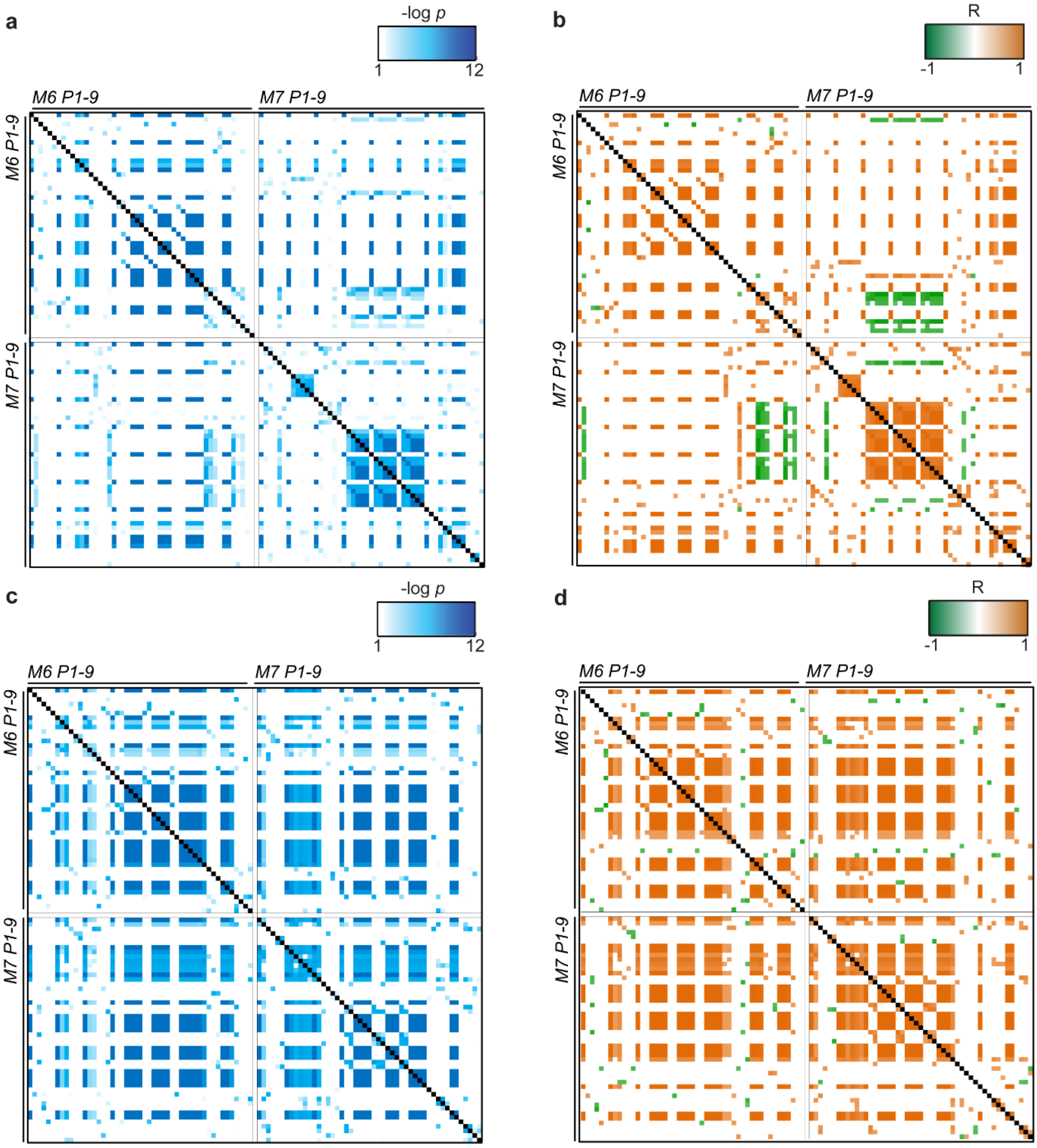

Suppl. Figure 4. Consistency analysis of time-dependent whole blood thrombus formation on GPVI-dependent surfaces. Whole blood from control subjects (n = 10) was flowed at 1000 s^{-1} over microspots *M6* (collagen-I ± TF) or *M7* (GFOGER-GPO ± TF). Parameter values (*P1-9)* were univariate scaled values across microspots. (A, C) Shown are *p* values of correlation analysis of microspots without (A) or with (C) co-coated TF. Blue colour intensity reflects $p = 10^{-1} – 10^{-12}$. Furthermore, R values of correlation analysis of microspots without (B) or with (D) TF. Only significant R values are shown (p<0.05). Full data are given in Data File 2.

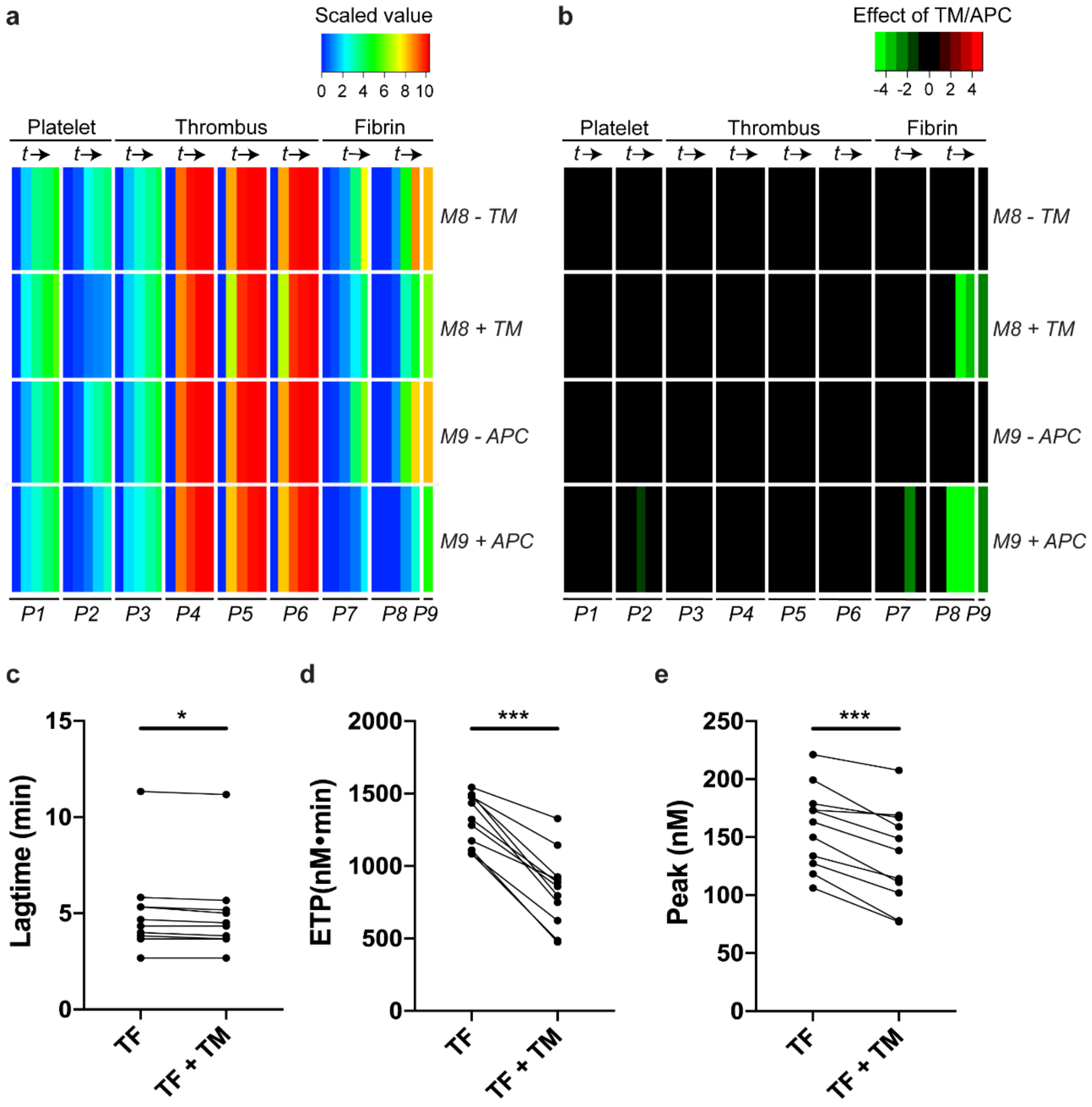

Suppl. Figure 5. Altered kinetics of whole blood thrombus by the protein C anticoagulation pathway. (A-B) Collagen-I microspots were co-coated or not with TM (*M8*) or APC (*M9*), and subjected to whole-blood flow at 1000 s^{-1} (see Figure 4). (A) Heatmap of parameters *P1-9* over time (→) for *M8-9* with or without TM or APC. Univariate scaling per parameter across surfaces; *n* = 10-11. (B) Subtraction heatmap indicating effects of co-coated TM or APC. Green (negative) and red (positive) colours indicate relevant changes (filtered for changes outside range of means ± SD). Full data are given in Data File 4. (C-E) Thrombin generation with platelet-poor plasma triggered with 1 pM TF, in the presence or absence of TM (0.325 nM). Effect of TM on thrombin lag time (C), endogenous thrombin potential (ETP) (D) and thrombin peak height (E). Dots represent values from individual control subjects.

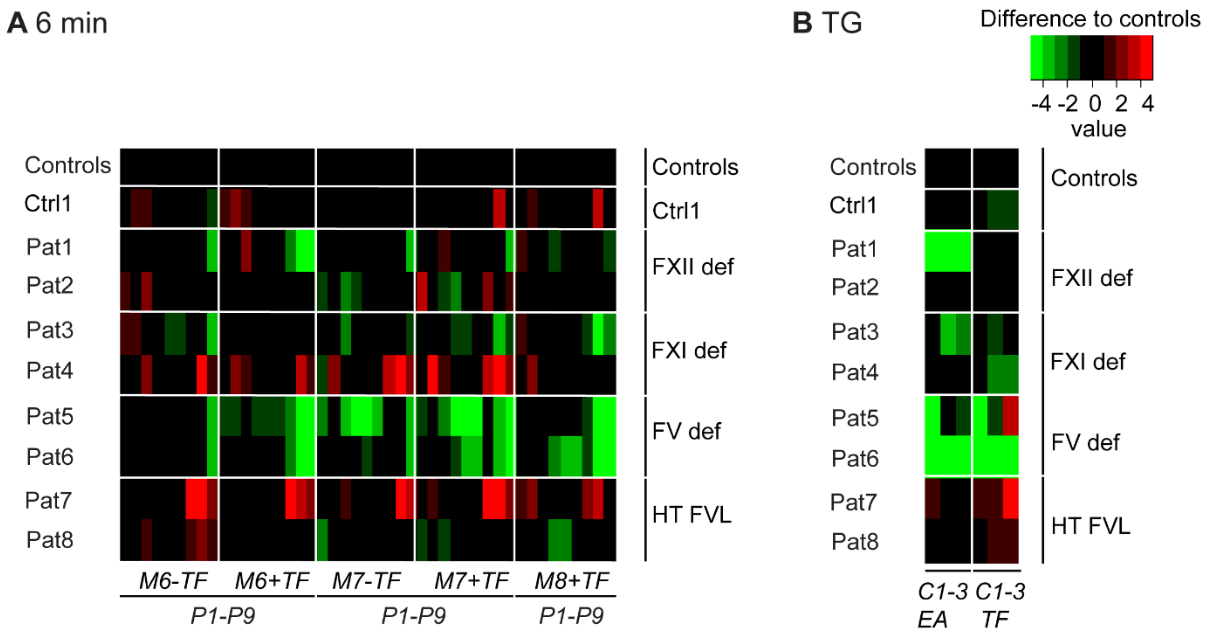

Suppl. Figure 6. Whole blood thrombus formation and thrombin generation for patients with coagulation factor abnormalities. Blood samples from healthy control subjects (*n* = 10) and patients (Pat, *n* = 8) with indicated coagulation factor abnormalities were analysed for kinetics of platelet-fibrin thrombus formation, as in Figure 5. Data per parameter were filtered for relevant increases (red) or decreases (green), when outside range of means ± SD of the control subjects. (A) Differential heatmap in comparison to control samples of scaled parameters per patient at *t* = 6 minutes. (B) Calibrated automated thrombin generation (CAT) measured in plasmas from control subjects and patients. Triggering was with ellagic acid (EA) or TF, and curve characteristics *C1-3* were measured (see Table 1).

Supplementary data files

Data File 1. Primary data of thrombus formation parameters for microspots *M1-7* with(out) tissue factor (Figure 3).

Data File 2. Primary *p* and *R* values of correlation analysis between parameters of thrombus formation (Figure 4).

Data File 3. Primary data of effects of CTI and/or iFVIIa on thrombus formation parameters for microspots *M1-7* (Figure 5).

Data File 4. Primary data of thrombomodulin and APC effects on thrombus formation parameters (Suppl. Figure 4).

Data File 5. Primary data of parameters of thrombus formation of patient blood samples (Figure 8).
